# Facile Fabrication of Novel NiFe_2_O_4_@Carbon Composites for Enhanced Adsorption of Emergent Antibiotics

**DOI:** 10.3390/ma14216710

**Published:** 2021-11-08

**Authors:** Van Tan Lam, Thi Cam Quyen Ngo, Long Giang Bach

**Affiliations:** 1 Institute of Environmental Technology and Sustainable Development, Nguyen Tat Thanh University, Ho Chi Minh City 700000, Vietnam; ntcquyen@ntt.edu.vn; 2 Department of Science and Technology, People’s Committee in Ben Tre, Ben Tre City 86000, Vietnam; 3 Faculty of Environment and Food Technology, Nguyen Tat Thanh University, Ho Chi Minh City 700000, Vietnam

**Keywords:** wastewater treatment, antibiotic pollutants, metal-organic frameworks, magnetic composites, response surface methodology

## Abstract

Water purification is becoming one of the most pertinent environmental issues throughout the world. Among common types of water pollution involving heavy metals, pharmaceutical drugs, textile dyes, personal care products, and other persistent organic pollutants, the pollution of antibiotic drugs is increasingly emerging due to their adverse effects on microorganisms, aquatic animals, and human health. Therefore, the treatment of such contaminants is very necessary to reduce the concentration of antibiotic pollutants to permissible levels prior to discharge. Herein, we report the use of NiFe_2_O_4_@C composites from a bimetallic-based metal-organic framework Ni-MIL-88B(Fe) for removal of ciprofloxacin (CFX) and tetracycline (TCC). The effect of production temperatures (600–900 °C), solution pH (2–10), NiFe_2_O_4_@C dose (0.05–0.2 g/L), concentration of antibiotics (10–60 mg/L), and uptake time (0–480 min) was investigated systematically. Response surface methodology and central composite design were applied for quadratic models to discover optimum conditions of antibiotic adsorption. With high coefficients of determination (R^2^ = 0.9640–0.9713), the proposed models were significant statistically. Under proposed optimum conditions, the adsorption capacity for CFX and TCC were found at 256.244, and 105.38 mg/g, respectively. Recyclability study was employed and found that NiFe_2_O_4_@C-900 could be reused for up to three cycles, offering the potential of this composite as a good adsorbent for removal of emergent antibiotics.

## 1. Introduction

Global industrialization not only accompanies drastically increasing demands for clean water, but it can also trigger many potential pollutions related to water resources. Therefore, water purification is currently one of the most pertinent environmental issues throughout the world. There are common types of water pollution caused by heavy metals, pharmaceutical drugs, textile dyes, personal care products, or other persistent organic pollutants [1,2,3]. Among them, the pollution of pharmaceutical drugs, including antibiotics, is alarmingly emerging due to their adverse effects on microorganisms, aquatic animals, and human health through bioaccumulation in food chains [4]. Tetracycline (TCC, molecular formula: C_22_H_24_N_2_O_8_, molar weight: 444.435 g/mol) acts as a broad-spectrum antibacterial antibiotic [5]. It has been widely consumed in livestock industries and human therapies mainly due to low-cost production [6]. It was estimated that TCC accounts for one third of the total production, and is ranked second in the consumption of worldwide antibiotics [7]. Meanwhile, ciprofloxacin (CFX, molecular formula: C_17_H_18_FN_3_O_3_, molar weight: 331.346 g/mol) presents as one of the most widely used second-generation quinolones [8]. It was reported that millions of prescriptions were consumed for antibacterial purposes, eye irritations, and bone infections in the US [9]. The occurrence of TCC and CFX pollution can originate from the wastewater discharge of pharmaceutical factories, or residuals of aquafarming activities. In hospital wastewaters, an amount of these pharmaceuticals can exist through untreated urine excretion as a result of partial metabolism in human bodies [10]. They directly enter and accumulate in water sources, enhancing the antibiotics-resistant activities of bacteria, and imposing great threats on human health and safety [11]. More importantly, both TCC and CFX exhibit a difficult degree of biodegradability in aquatic systems [12,13]. Hence, adoption of treatment technologies is wholly necessary to reduce the concentration of TCC and CFX antibiotics to permissible levels prior to discharge.

There are a variety of physical and chemical techniques applied for the treatment of TCC and CFX antibiotics. For example, filtration and infiltration processes using granulation, activated alumina, and activated carbon for water purification have been addressed [14,15]. However, many studies indicated that adsorption acted as a feasible approach [16]. Indeed, the main advantages of adsorption such as simplicity, outstanding recyclability, and high efficiency of removal of these pharmaceuticals and others, were demonstrated [17,18]. Various materials such as metal-organic frameworks [19], ferrites [20], composite carbon nanotubes [21], activated carbons [22], and functionalized zeolites [23] have been studied as promising adsorbents. Among the materials mentioned above, ferrites such as Fe_3_O_4_, NiFe_2_O_4_, CoFe_2_O_4_, and MnFe_2_O_4_ possess a special magnetic property, which enables easy separation from the solutions. Previous literatures have addressed the use of NiFe_2_O_4_ ferrites in wastewater remediation [24,25,26]. However, the adsorption efficiency of single ferrites is relatively low, and its recyclability seems unstable. Porous carbons have superior advantages, i.e., their structure often obtains high porosity, and surface area, along with an amount of functional groups, which accelerates the efficiency of antibiotics removal [27,28]. Attachment of magnetic components (e.g., NiFe_2_O_4_) on porous carbon structure is highly expected to reach out higher results of uptake effectiveness and separation. Thus, fabrication of such magnetic porous carbons has recently attracted great attention [29,30,31]. Several methods such as coprecipitation and impregnation have been widely applied, but many disadvantages (e.g., poor dispersion of ferrite nanoparticles in carbon matrixes or layers due to magnetic agglomeration) were observed [32,33,34]. These problems can be solved by using metal-organic frameworks (MOFs) as self-sacrificial templates through pyrolysis to synthesize magnetic carbons. After the carbonization process, the crystalline structure of MOFs including both metal clusters and ligands is decomposed; leaving a carbon skeleton embedded with metal oxides [35,36]. This strategy was successfully applied to form magnetic composite carbons in our previous studies [37,38,39].

In this study, we converted Ni-MIL-88B(Fe) as a template into novel nickel ferrites-embedded porous carbons (NiFe_2_O_4_@C). Various temperatures at 600, 700, 800, and 900 °C were investigated. The composite materials were characterized and applied as adsorbents for the adsorption of TCC and CFX antibiotics in waters. The effect of contact time, antibiotics concentration, solution pH, and NiFe_2_O_4_@C dosage was surveyed. Adsorption kinetics and isotherms for antibiotic uptake on NiFe_2_O_4_@C were also performed herein. To the best of our knowledge, there was still no report on the use of NiFe_2_O_4_@C adsorbents derived from Ni-MIL-88B(Fe) for TCC and CFX antibiotics removal.

## 2. Experimental

### 2.1. Chemicals

In this study, tetracycline hydrochloride, ciprofloxacin, 1,4–benzene–dicarboxylic acid (H_2_BDC), iron(III) nitrate nonahydrate (Fe(NO_3_)_3_∙9H_2_O), nickel(II) chloride hexahydrate (NiCl_2_∙6H_2_O), *N,N*–dimethylformamide (DMF), triethylamine, methanol (CH_3_OH), and acetonitrile (CH_3_CN) were commercially purchased from Sigma–Aldrich. These chemicals were used without any need of purification. Double-distilled water was used to dilute the aqueous solutions.

### 2.2. Instrumentation

The D8 Advance Bruker powder diffractometer was used to record the X-ray powder diffraction (XRD, Hitachi Inc., Krefeld, Germany) profiles using Cu–Kα beams as excitation sources. The S4800 instrument (JEOL, Tokyo, Japan) was implemented to capture the scanning electron microscope (SEM) images with magnification of 7000× using an accelerating voltage source (15 kV). The N_2_ adsorption/desorption isotherm measurements were recorded on the Micromeritics 2020 volumetric adsorption analyzer system (Micromeritics Inc., Norcross, GA, USA). The FT-IR spectra were recorded on the Nicolet 6700 spectrophotometer (Thermo Fischer Scientific Inc., Waltham, MA, USA). The UV–Vis spectrophotometer (Shimadzu, Kyoto, Japan) was used to determine the concentrations of TCC and CFX antibiotics at 273 and 272 nm in wavelength, respectively.

### 2.3. Synthesis of Ni-MIL-88B(Fe) Material

**Figure 1:**
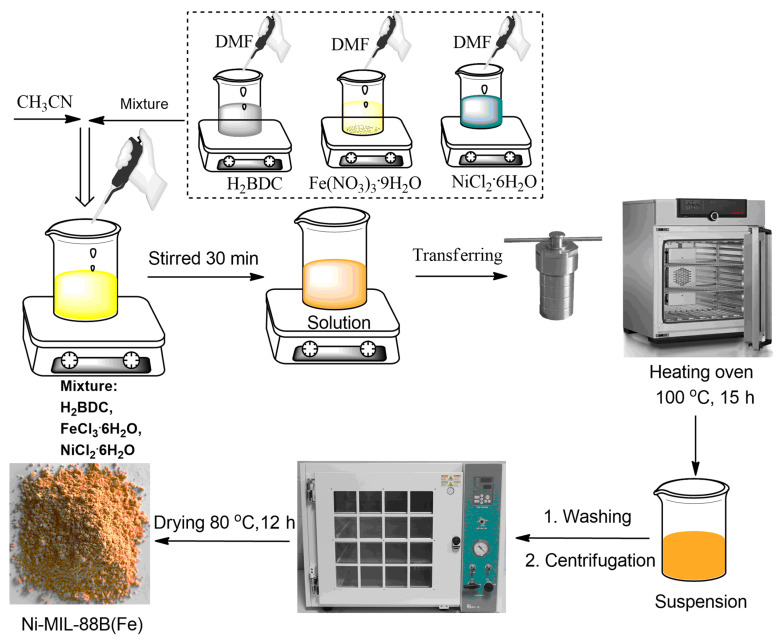
Schematic route for the synthesis of Ni-MIL-88B(Fe).

The Ni-MIL-88B(Fe) material could be fabricated by solvothermal method, referring to a previous study with some modifications of amount of salts and solvents [40]. In detail, the synthesis process is summarized in Figure 1. As a typical procedure, 0.3 g H_2_BDC, 0.4309 g Fe(NO_3_)_3_·9H_2_O, and 0.1268 g NiCl_2_·6H_2_O were each dissolved completely in separate beakers containing DMF (40 mL) under stirring for 15 min. All solutions were then poured into a larger beaker, and CH_3_CN (40 mL) was carefully added under stirring for the next 30 min. The homogeneous mixture was divided and transferred into Teflon-lined autoclaves with appropriate volume. All autoclaves were loaded in a heating oven at 100 °C for 15 h. After cooling down the oven, the suspension was extracted, washed with DMF (three times) and methanol (three times) to make sure that all residual metals and impurities were removed. The solid was separated from the solution by centrifugation. Finally, all bold red yellow crystals were dried by vacuum system and stored for usage as template precursor for NiFe_2_O_4_@C synthesis.

### 2.4. Synthesis of NiFe_2_O_4_@C Materials from Ni-MIL-88B(Fe)

As-produced Ni-MIL-88B(Fe) was used as a template to synthesize NiFe_2_O_4_@C. As a typical procedure, 2.0 g Ni-MIL-88B(Fe) were heated (ramping rate of 3 °C/min) at various temperatures, 600, 700, 800, and 900 °C, from the room temperature for 4 h. To make sure that degasification of oxygen was complete, nitrogen (100 mL/min) was flowed continuously throughout the samples for 1 h before pyrolysis. After pyrolysis, the system temperature was allowed to reduce gradually to room temperature beneath nitrogen flow (100 mL/min). The pyrolysis products were collected and labeled as NiFe_2_O_4_@C-x, where x denotes the pyrolysis temperature (NiFe_2_O_4_@C-600, NiFe_2_O_4_@C-700, NiFe_2_O_4_@C-800, and NiFe_2_O_4_@C-900).

### 2.5. Adsorption Experiments

All the adsorption experiments were carried out under batch mode at room temperature, except for further indication. Each of the adsorbents, including Ni-MIL-88B(Fe) and NiFe_2_O_4_@C-x (0.05–0.2 g/L), were weighed, and loaded into 250-mL beakers containing TCC or CFX solutions (50 mL) at various concentrations (5–60 mg/L), and pH (2–10). After adsorption for 480 min, the antibiotic solution was sampled, and measured by UV–Vis spectroscopy to determine the concentration. For kinetic experiments, the aliquot (2 mL) was sampled at various interval periods (0, 5, 10, 20, 30, 60, 90, 120, 180, 240, 360, and 480 min). The percentage of removal (*H*, %) and uptake capacity (*q_e_*, mg/g) were defined by Equations (1) and (2).

(1) $$H\left(\%\right)=\frac{C_{0}-C_{f}}{C_{0}}\times 100$$

(2) $$q_{e}=\frac{C_{0}-C_{f}}{W}\times V$$

where, *C*_0_ (mg/L) and *C_f_* (mg/L) denote the initial and finial concentrations, respectively; *W* (g) and *V* (L) denote the mass of adsorbent and volume of the solution, respectively.

### 2.6. Experimental Design for Optimization

After analyzing the most influential effect of a range of factors, three main factors involving antibiotic concentration, sorbent mass, and pH were selected to discover the optimum adsorption of antibiotic ciprofloxacin (CFX) and antibiotic tetracycline (TCC). The response surface methodology was applied for statistical analysis, and proposed solutions to reach the highest adsorption capacity. In detail, based on the Box–Behnken design, Table 1 summaries the list of factors and value ranges for adsorption models of TCC and CFX on adsorbent.

## 3. Results and Discussion

### 3.1. Characterization

#### 3.1.1. XRD Analysis

**Figure 2:**
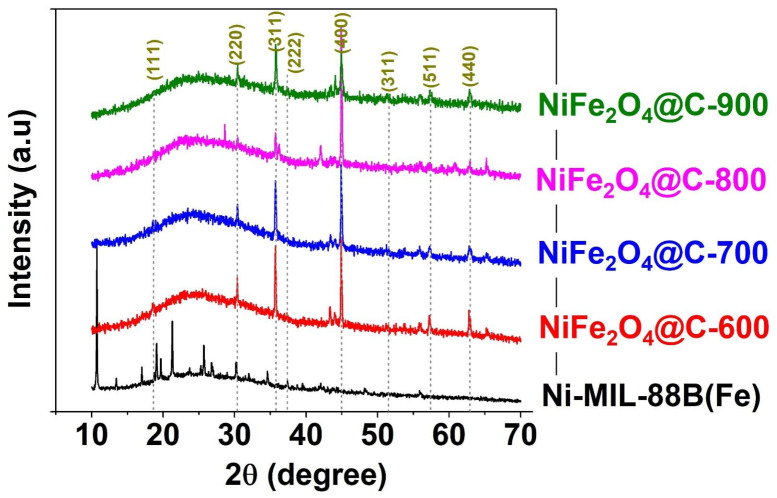
XRD pattern profiles of Ni-MIL-88B(Fe), NiFe_2_O_4_@C-600, NiFe_2_O_4_@C-700, NiFe_2_O_4_@C-800, and NiFe_2_O_4_@C-900 materials.

Figure 2 presents the results of crystalline structural analysis of Ni-MIL-88B(Fe), NiFe_2_O_4_@C-600, NiFe_2_O_4_@C-700, NiFe_2_O_4_@C-800, and NiFe_2_O_4_@C-900 materials through X-ray diffraction patterns. For Ni-MIL-88B(Fe), the main peaks appear at positions 10°, 16.8°, 17.7°, 18.7°, 20.1°, and 22°, which corresponded to that of past literature [41]. This material has a high crystallinity, indicating that it has been successfully synthesized by solvothermal method. Meanwhile, the crystal profile of pyrolysis materials as NiFe_2_O_4_@C-x (x = 600, 700, 800, 900) were completely different from that of the precursor, offering the event that Ni-MIL-88B(Fe) was decomposed under the effect of thermal energy. However, many typical peaks at 18.7° (111), 30.4° (220), 35.8° (311), 37.4° (222), 44.9° (400), 51.6° (311), 57.4° (511), and 63.0° (440) supported the evidence of single-phase cubic spinel NiFe_2_O_4_ in composite structure of NiFe_2_O_4_@C-x. This finding fitted very well with the XRD standard profile of pure NiFe_2_O_4_ (JCPDS PDF No. 10-0325), as well as many reported documents [42,43]. Moreover, graphitic carbon was found due to the presence of a broad peak between 20 and 30°. This crystallite phase may be formed by the destruction of ligand structure containing aromatic rings, converting them into carbon layers or matrix. A previous study reported the same phenomenon caused by carbonizing metal-organic framework under nitrogen to break down the coordination bonds, and form ionic bonds as of NiFe_2_O_4_ [44]. The existence of the magnetic component aids the separation of the sorbent materials while the porous carbon component may enhance the adsorption of antibiotic drugs.

#### 3.1.2. FT–IR Analysis

**Figure 3:**
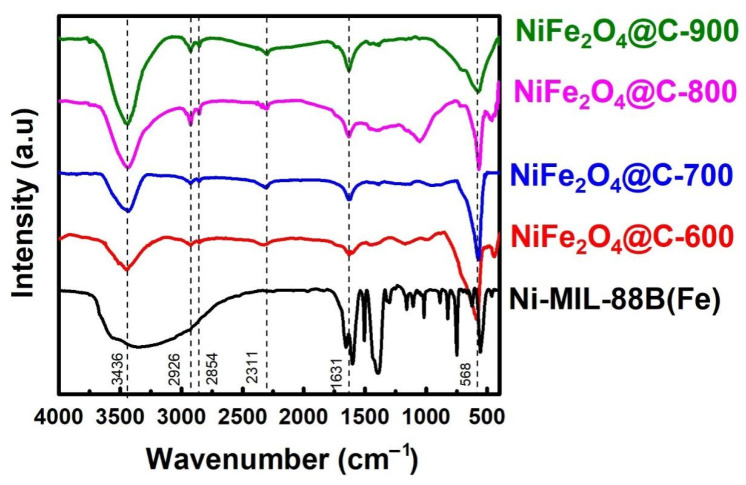
FT–IR spectra of Ni-MIL-88B(Fe), NiFe_2_O_4_@C-600, NiFe_2_O_4_@C-700, NiFe_2_O_4_@C-800, and NiFe_2_O_4_@C-900 materials.

The FT-IR spectra presented in Figure 3 show the moderate difference between original Ni-MIL-88B(Fe) and NiFe_2_O_4_@C-x materials. Overall, there are several peaks that still remained, but others are absent from the original structure. In detail, a strong absorption band at the wavenumber 3500–3750 cm^−1^ was typical for the –OH functional groups in all samples [45]. Another absorption band at 1600–1631 cm^−1^ was maintained, and characteristic of the vibration of C=O bonds, regardless of a minor shift of wavenumber from 1600 in Ni-MIL-88B(Fe) to 1631 cm^−1^ in NiFe_2_O_4_@C-x [46]. The strong emerging absorption band at 1392 cm^−1^ characterized the symmetrical and asymmetrical oscillations of carboxylate groups (–COO) of ligand on Ni-MIL-88B(Fe), but it was not observed on NiFe_2_O_4_@C-x series [47]. This finding provided the information of breaking out the carboxylate ligands under pyrolysis. Clear evidence of the presence of C–H at 2926, 2864, and 750 cm^−1^ was presented in NiFe_2_O_4_@C-x materials [48]. These chemical bonds were typical on graphical carbon with high hydrophobicity. The peaks shifting from 558 to 568 cm^−1^ were observed since the temperature of pyrolysis increased and it was attributable to the existence of Fe–O [49,50]. This proof consolidated the successful conversion of coordination bonds of Ni-MIL-88B(Fe) into ionic bonds of NiFe_2_O_4_@C-x. It is possible that carbon acts a strong reductant to transform Fe(III) into Fe(II) species. The previous study explained the same event occurred on MIL-53(Fe) or Fe(BDC) (BDC = 1,4-benzene-dicarboxylic acid) material [51].

#### 3.1.3. Morphological Analysis

**Figure 4:**
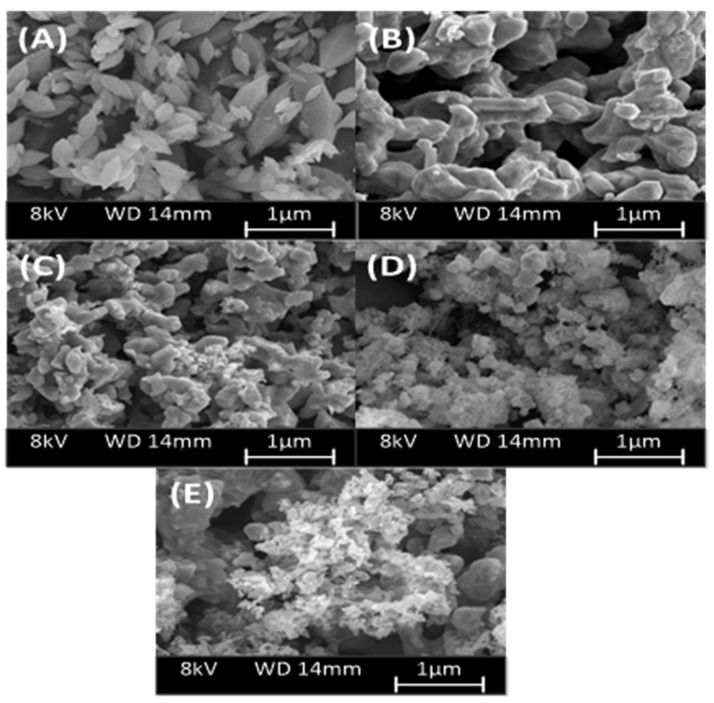
SEM microphotography of (**A**) Ni-MIL-88B(Fe), (**B**) NiFe_2_O_4_@C-600, (**C**) NiFe_2_O_4_@C-700, (**D**) NiFe_2_O_4_@C-800, and (**E**) NiFe_2_O_4_@C-900 materials.

Morphological characteristics of original Ni-MIL-88B(Fe) and NiFe_2_O_4_@C-x materials by scanning electron microscopy (SEM) analysis. Figure 4A shows that Ni-MIL-88B(Fe) particles have a relatively uniform size distribution with mainly hexagonal shape. This observation was well consistent with that of a previous study [40]. When carbonizing Ni-MIL-88B(Fe) material at different temperatures, the morphology of the pyrolysis materials changes markedly as shown in Figure 4B–E. The results indicate that the morphology of NiFe_2_O_4_@C-x became relatively amorphous, and their surface has more defects at higher calcination temperature. This finding was explained due to the effect of thermal energy, causing the destruction of hexagonal structure, and strong rearrangement of metal atoms under magnetic field.

#### 3.1.4. Nitrogen Adsorption/Desorption Isotherm Analysis

**Figure 5:**
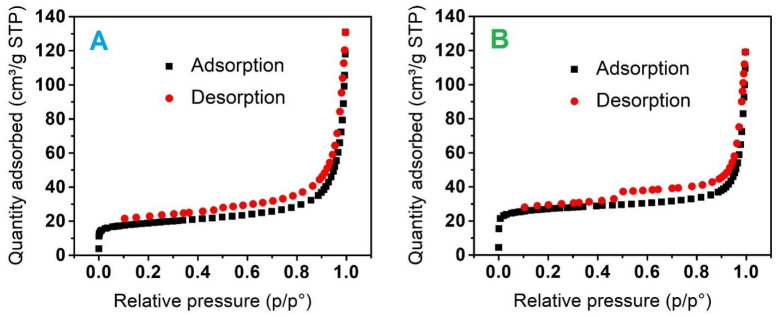
Nitrogen adsorption–desorption isotherm of (**A**) Ni-MIL-88B(Fe) and (**B**) NiFe_2_O_4_@C-900 materials.

Ni-MIL-88B(Fe) and NiFe_2_O_4_@C-900 were selected as representatives to characterize the nitrogen adsorption and desorption isotherms as shown in Figure 5. The results depict that the isotherms resemble between mixed Type II and Type IV curves with the presence of hysteresis, indicating that the structures of Ni-MIL-88B(Fe) and NiFe_2_O_4_@C-900 obtained mesoporous and microporous properties. In addition, BET surface area and pore volume of NiFe_2_O_4_@C-900 were measured, at 68.3 m^2^/g, and 0.1541 cm^3^/g, respectively, which were considerably lower than those of original Ni-MIL-88B(Fe), at 268 m^2^/g, and 0.14 cm^3^/g, respectively. This finding reconfirmed that the pyrolysis process causes the collapse of crystalline structure, and agglomeration of magnetic NiFe_2_O_4_ nanoparticles during pyrolysis at high temperature (900 °C). However, NiFe_2_O_4_@C-900 with a sufficiently large surface area and diverse surface groups can attain good adsorption efficiency results.

### 3.2. Adsorption Study

#### 3.2.1. Effect of Pyrolysis Temperature

**Figure 6:**
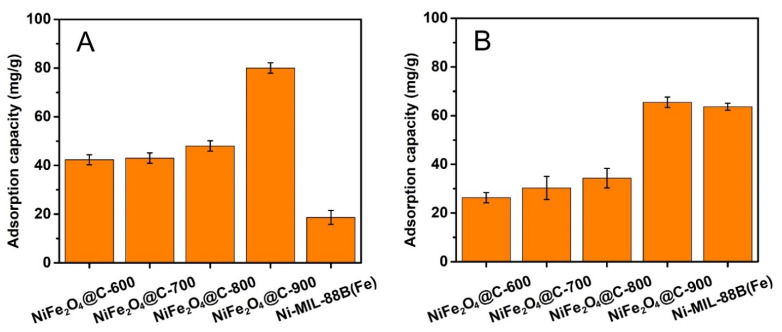
CFX (**A**) and TCC (**B**) antibiotic adsorption capacities of Ni-MIL-88B(Fe), NiFe_2_O_4_@C-600, NiFe_2_O_4_@C-700, NiFe_2_O_4_@C-800, and NiFe_2_O_4_@C-900 materials. Experimental conditions included contact time: 240 min, dose: 0.1 g/L, initial concentration (CFX and TCC): 20 mg/L, temperature: 30 °C, pH: unadjusted, repeatability (*n* = 3) and error bars for all experiments.

Calcination condition strongly affects the structure and adsorption properties of materials. Thus, the composite materials produced at various temperature were compared in adsorption capacity to CFX and TCC. Figure 6 shows that NiFe_2_O_4_@C-900 achieved the highest adsorption capacity in both CFX and TCC antibiotics, 80.0 mg/g and 65.5 mg/g, respectively. This can be explained as follows. At higher pyrolysis temperatures, the structures of NiFe_2_O_4_@C-x are more defective, higher degree of graphitic carbons, and larger number of functional groups. Therefore, the adsorption capacity of NiFe_2_O_4_@C-x was higher at the higher produced temperatures.

Herein, Ni-MIL-88B(Fe) attained a relatively good adsorption capacity to TCC antibiotic (63.7 mg/g), which was very close to that of NiFe_2_O_4_@C-900. However, this material exhibited some disadvantages such as poor stability in aqueous solvent (particularly at low pH), difficult recovery after treatment, and low recyclability. As a result, Ni-MIL-88B(Fe) was not selected to perform further investigations.

#### 3.2.2. Effect of Solution pH

**Figure 7:**
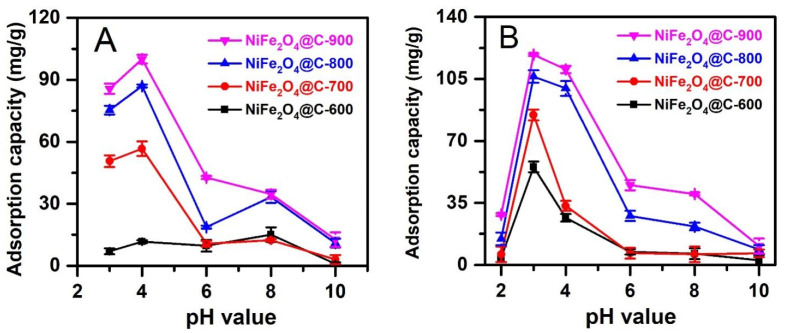
Effect of pH values (2–10) on CFX (**A**) and TCC (**B**) adsorption capacity of NiFe_2_O_4_@C-600, NiFe_2_O_4_@C-700, NiFe_2_O_4_@C-800, and NiFe_2_O_4_@C-900 materials. Experimental conditions included contact time: 240 min, dose: 0.1 g/L, initial concentration (CFX and TCC): 20 mg/L, temperature: 30 °C, pH: 2, 3, 4, 6, 8, and 10, repeatability (*n* = 3) and error bars for all experiments.

Surface charge of magnetic composites and ionization of antibiotic molecules can be controlled by pH value. Thus, the influence of solution pH 2–10 on CFX and TCC adsorption capacity was investigated in this work. Figure 7 shows the highest adsorption capacity of CFX and TCC on NiFe_2_O_4_@C-x series at pH 4 and pH 3, respectively, except for the case of CFX adsorption by NiFe_2_O_4_@C-600, where optimum pH was found at 8.0. NiFe_2_O_4_@C-900 exhibited the highest CFX and TCC adsorption capacities, at 100.0 mg/g and 118.7 mg/g, respectively. These optimum pH values found herein were consistent with some previous studies [12,52]. Therefore, the CFX solution at pH 4, and TCC solution at pH 3 were selected to investigate for the effect of adsorbent dose.

#### 3.2.3. Effect of Composite Dose

**Figure 8:**
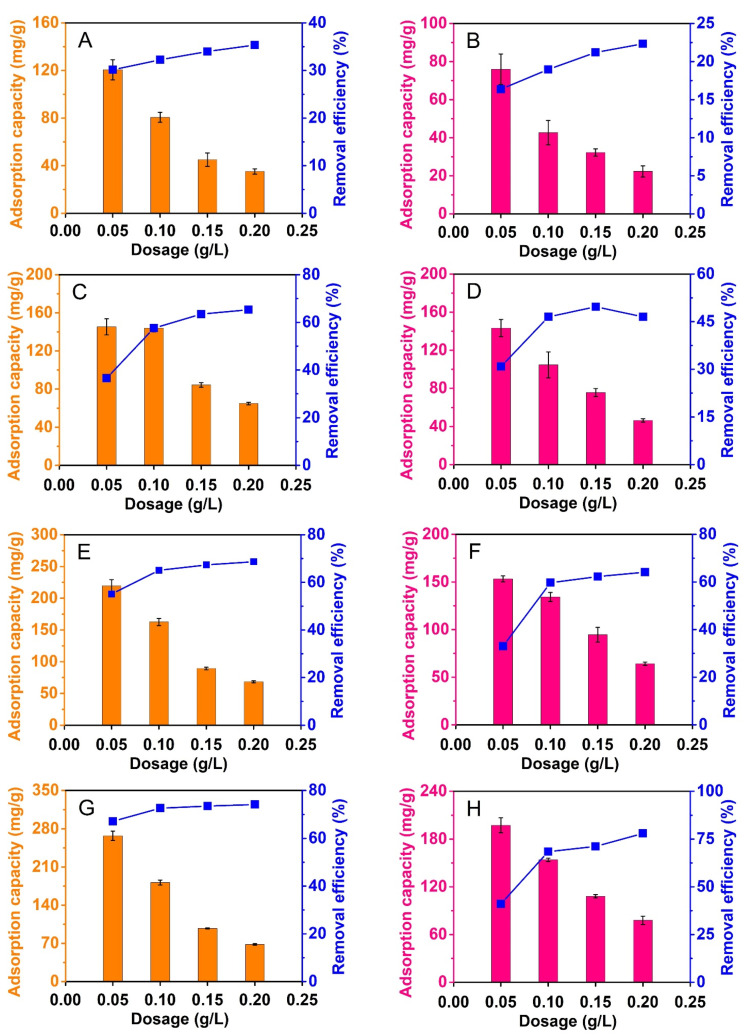
Effect of adsorbent dosages (0.05–0.2 g/L) on CFX (**A**,**C**,**E**,**G**) and TCC (**B**,**D**,**F**,**H**) adsorption of NiFe_2_O_4_@C-600 (**A**,**B**), NiFe_2_O_4_@C-700 (**C**,**D**), NiFe_2_O_4_@C-800 (**E**,**F**), and NiFe_2_O_4_@C-900 (**G**,**H**) materials. Experimental conditions included contact time: 240 min, dose: 0.05, 0.1, 0.15, and 0.2 g/L, initial concentration (CFX and TCC): 20 mg/L, temperature: 30 °C, pH: 4 (for CFX) and 3 (for TCC), repeatability (*n* = 3) and error bars for all experiments.

Adsorbent dosage is optimized to reach lower amounts of used materials, but higher level of antibiotic treatment. In this study, composite dosage was set between 0.05 and 0.2 g/L. The adsorption capacity and removal efficiency were measured to totally assess the effect of adsorbent dosage. Figure 8 indicates that composite dose has strong effects on adsorption of CFX and TCC. Overall, the dose increased from 0.05 to 0.2 g/L, the removal efficiency would gradually increase, but uptake capacity would gradually decrease in most cases. These findings are because higher dose of materials provided more adsorptive sites to improve the percentage of antibiotic removal. However, the use of higher amount of composite could lead to a decrease of uptake capacity based on Equation (2). The same phenomenon of adsorption of CFX and TCC on various composites was found in the previous studies [53,54].

In particular, the adsorbents produced at higher temperatures gave higher adsorption of both CFX and TCC. Indeed, take dose of 0.1 g/L as an example, the percentages of CFX and TCC removal by NiFe_2_O_4_@C-600 obtained at only 32.3% and 19%, respectively, but those by NiFe_2_O_4_@C-900 reached the highest values, at 72.7% and 68.4%, respectively. At the same trend, uptake capacity of CFX and TCC by NiFe_2_O_4_@C-600 obtained at only 80.7 mg/g and 42.7 mg/g, respectively, but those by NiFe_2_O_4_@C-900 reached the highest values, at 181.7 mg/g and 154 mg/g, respectively. This was due to pyrolysis conditions’ influence on pore structure and surface area of composites. Finally, we considered several factors of material mass as well as adsorption efficiency to select a dose of 0.1 g/L for further investigations.

#### 3.2.4. Effect of Contact Time

**Figure 9:**
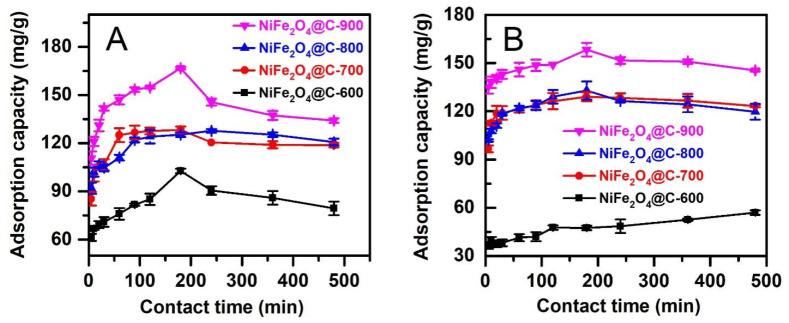
Effect of contact times (0–480 min) on adsorption capacity on CFX (**A**) and TCC (**B**) adsorption on NiFe_2_O_4_@C-600, NiFe_2_O_4_@C-700, NiFe_2_O_4_@C-800, and NiFe_2_O_4_@C-900 materials. Experimental conditions included contact time: 0–480 min, dose: 0.1 g/L, initial concentration (CFX and TCC): 20 mg/L, temperature: 30 °C, pH: 4 (for CFX) and 3 (for TCC), repeatability (*n* = 3) and error bars for all experiments.

Figure 9 shows the amount of CFX and TCC antibiotics adsorbed on NiFe_2_O_4_@C-x composite against contact time. In general, the adsorption occurred rapidly at the initial periods (0–30 min) due to the availability of adsorptive sites. Most of the kinetic adsorption curves reached a threshold of equilibrium at about 180 min. Adsorption CFX and TCC antibiotics by NiFe_2_O_4_@C-x was unconducive after this moment because desorption was initiated. This event was because the water molecules would enhance the occupation of pores of NiFe_2_O_4_@C-x, pushing the antibiotic molecules away from the adsorbents. Moreover, the immersion of composite adsorbent in aqueous solutions reduced their stability. Based on such observations, optimum time of 180 min was selected to explore the effect of further parameters such as antibiotic concentration.

According to Figure 9, NiFe_2_O_4_@C-900 gave the highest removal percentage and adsorption capacity. Therefore, this material was used to calculate the kinetic constants from pseudo first-order, pseudo second-order, Elovich, and Bangham. Table 2 compares the fitness of kinetic models by coefficient of determination (R^2^). With R^2^ values greater than 0.9, adsorption kinetics described the experimental data well. TCC adsorption was followed by both Elovich and Bangham, while CFX adsorption was followed by pseudo second–order equation.

#### 3.2.5. Effect of Initial Concentration

**Figure 10:**
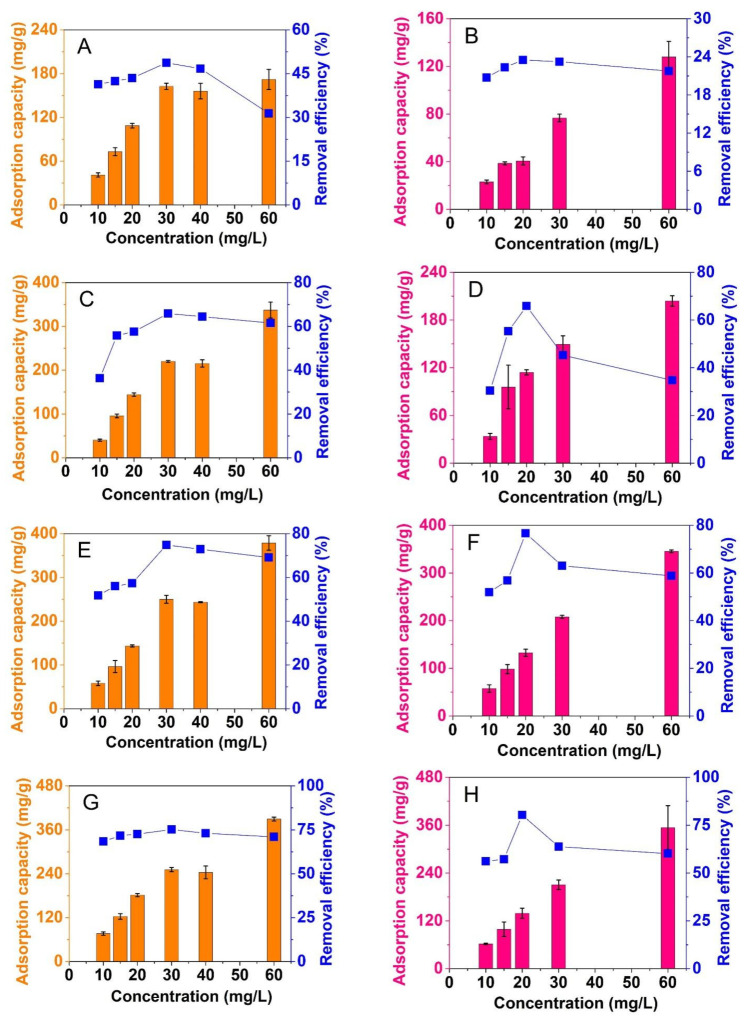
Effect of initial CFX (**A**,**C**,**E**,**G**) and TCC (**B**,**D**,**F**,**H**) concentrations (10–60 mg/L) on adsorption capacity and removal efficiency on NiFe_2_O_4_@C-900. Experimental conditions included contact time: 180 min, dose: 0.1 g/L, initial concentration (CFX and TCC): 10, 15, 20, 30, 40, and 60 mg/L, temperature: 30 °C, pH: 4 (for CFX) and 3 (for TCC), repeatability (*n* = 3) and error bars for all experiments.

Figure 10 shows the amount of CFX and TCC antibiotics adsorbed on NiFe_2_O_4_@C-x composite against initial concentration. In general, the adsorption capacity would increase with increasing concentration of antibiotics. The composite adsorbent produced at higher temperature gave better results of uptake capacity. NiFe_2_O_4_@C-900 gave the highest adsorption capacity for CFX (389 mg/g) and TCC (354 mg/g) at initial concentration of 60 mg/L. At the same trend, this material gave the highest percentage of removal for CFX (75.2%) and TCC (76.7%) at initial concentration of 30 mg/L and 20 mg/L, respectively.

To gain insight into the adsorption equilibrium, the isotherm equations including Langmuir, Freundlich, and Temkin were fitted using their nonlinear forms. Table 3 lists the isotherm constants for adsorption of CFX and TCC on NiFe_2_O_4_@C-900 material. Based on R^2^ values, the best fitting order of CFX adsorption isotherm models would be: Temkin > Langmuir > Freundlich, and that of TCC adsorption isotherm models would be: Langmuir > Langmuir > Temkin. As a result, adsorption of CFX and TCC on NiFe_2_O_4_@C-900 followed by Temkin and Langmuir, respectively. With a range of values R_L_ and 1/n between zero and one, the adsorption of antibiotics in this study offered a favorable process. More importantly, the values of maximum adsorption capacity of CFX and TCC identified from Langmuir were very high, 737.42 mg/g and 827.34 mg/g, respectively. It is therefore proposed that NiFe_2_O_4_@C-900 can be a good adsorbent to adsorb CFX and TCC from water.

### 3.3. Adsorption Optimization Using Response Surface Methodology

#### 3.3.1. Model Design

From results obtained from investigating the effect of each factor, the optimization model was designed by selecting the powerful factors such as antibiotic concentration, adsorbent dosage, and pH. Based on the widely used central composite design, the range of experimental values are shown in Table 1. There are twenty experimental runs for each antibiotic adsorption model, giving twenty experimental results. The corresponding twenty predicted results were listed in Table 4. Overall, the adsorption capacity of CFX antibiotic reached the highest value at 285 mg/g (entry 10), and the lowest at 33.21 mg/g (entry 1). Meanwhile, TCC absorption capacity was the highest value of 105 mg/g (entry 16), and the lowest value of was 29 mg/g (entry 9).

The relationship between the antibiotics adsorption capacity on NiFe_2_O_4_@C-900 and independent variables x_1_, x_2_, and x_3_ generated from Design-Expert 11 software expressed in the actual equation is as follows:

Q_TCC_ (mg/g) = −120.61 + 9.89 x_1_ + 320.26 x_2_ + 55.84 x_3_ − 11.25 x_1_ x_2_ − 0.21 x_1_ x_3_ + 87.50 x_2_ x_3_ − 0.17 x_1_^2^ − 1421.04 x_2_^2^ − 9.21 x_3_^2^, (3)

Q_CFX_ (mg/g) = −1148.96 + 11.09 x_1_ + 2473.45 x_2_ + 470.99 x_3_ + 10.75 x_1_ x_2_ + 0.04 x_1_ x_3_ –357.50 x_2_ x_3_ − 0.12 x_1_^2^ − 5006.86 x_2_^2^ − 52.82 x_3_^2^, (4)

where x_1_ is the antibiotic concentration (mg/L), x_2_ is the adsorbent mass (g/L), and x_3_ is the pH value. From Equations (3) and (4), all factors had positive, and great effects on adsorption capacity, which means that the value of response would increase with the increase of factors. 

To assess the compatibility of each model, statistical analysis such as ANOVA (analysis of variance) discloses the significance based on the model terms such as sum of squares, degrees of freedom, mean of squares, F- and *p*-values, as well as fitness statistics. In detail, the statistically analytical results are summarized in Table 5.

According to Table 5, CFX and TCC antibiotic treatment models exhibit F-values of 29.74 and 37.57, respectively, corresponding to *p*-values less than 0.0001. This means that both models presented a chance of 0.01% that F-values could occur due to noise. As a result, both design models were statistically significant at a reliability level of 95%. More importantly, all factors which obtained *p*-value less than 0.0001, were statistically significant at the same reliability level. As such, the models have been successfully designed with the statistical significance of all factors [55]. 

Some fitness statistics such as coefficient of determination (R^2^) and adequate precision could be used to diagnose the compatibility of models. Obviously, R^2^ values for both models (0.9640–0.9713) were very close to 1.0, suggesting the outstanding fitness properties. In addition, both models had adequate precision (AP) values between 17.6179 and 19.9834. A model with AP value greater than 4.0 offers a high degree of goodness and compatibility. Here, AP values were both greater than 4.0, indicating the high compatibility of proposed models, providing the appropriate signals for the quadratic models [56]. The coefficient of variation (CV%) is a statistical measure of data dispersion level in a collection of data relative to the mean. As observed, both indicators (6.05–11.81%) were lower than 15%, suggesting that the dispersion of experimental data of proposed models should be acceptable.

**Figure 11:**
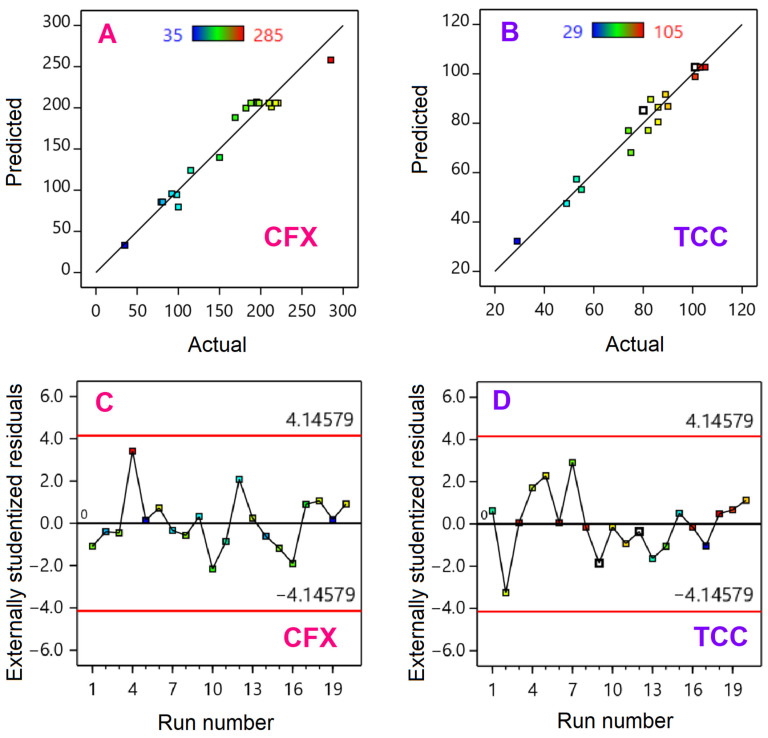
Actual versus predicted plots (**A**,**B**) and residuals versus runs plots (**C**,**D**) for CFX (**A**,**C**) and TCC (**B**,**D**) adsorption models.

Diagnostic plots can be used to evaluate whether a model is compatible with the experimental results. One of the most common ones is predicted versus actual plots as shown in Figure 11A,B. It was obvious that model data acquired a good correlation since the data points were concentrated in straight lines. Moreover, plots in Figure 11C,D again supported the evidence of random distribution of data points without any trends or scatters.

#### 3.3.2. Optimization of Process Parameters

**Figure 12:**
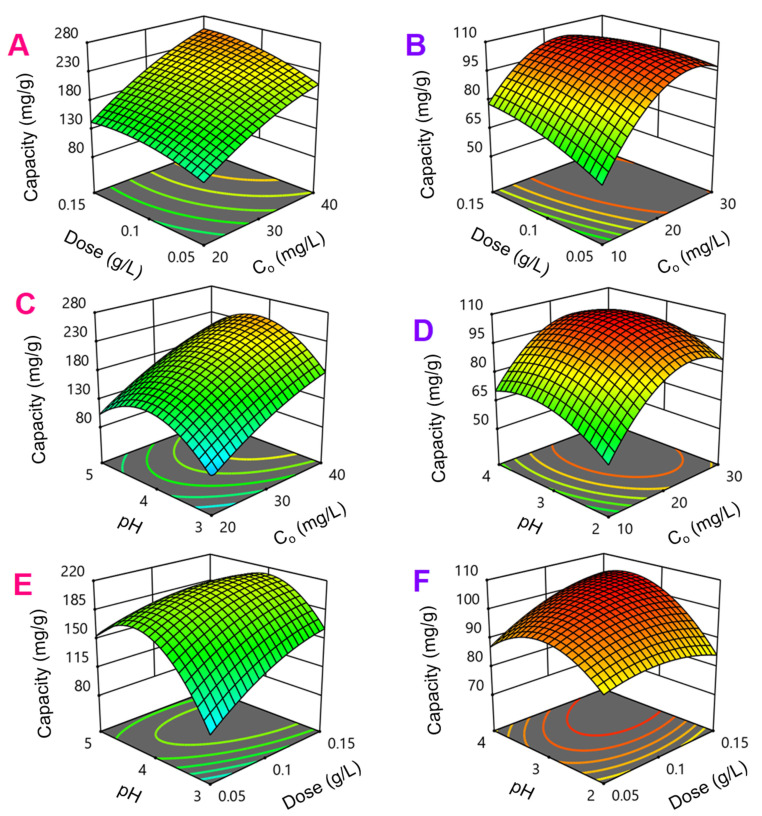
Response surfaces of CFX (**A**,**C**,**E**) and TCC (**B**,**D**,**F**) adsorption models.

The influence of influential factors on the adsorption capacity of TCC and CFX antibiotics by NiFe_2_O_4_@C-900 can be depicted by three-dimensional surface response plots as shown in Figure 12. Herein, two of three factors would be changed while another was kept at a central level or zero. Overall, the grid regions that described adsorption capacity were converged, indicating that the model could be optimized in the range of investigated values of factors. In detail, for response surfaces of the CFX adsorption model in Figure 12A,C,E, the adsorption capacity would be optimized at around pH 4. This finding was again compatible with surveyed results in the previous sections. The great improvement could be obtained if the CFX concentrations were set to high values (30–40 mg/L). NiFe_2_O_4_@C-900 adsorbent dose (0.05–0.15 g/L), however, showed a moderately influential effect, but an interactive correlation with other factors.

Meanwhile, for response surfaces of the TCC adsorption model, the response regions could be highly converged, which are highlighted with red color in Figure 12B,D,F. Blue color regions showed lower values of adsorption capacity. Based on optimum surfaces, the TCC concentrations were suggested from 20 to 30 mg/L to obtain higher magnitude of adsorption capacity. The conditions at around pH 3 would facilitate the adsorption of TCC, which was fitted with surveyed results in the previous sections. As similar as the case of CFX adsorption model, the effect of NiFe_2_O_4_@C-900 adsorbent dose (0.05–0.15 g/L) was moderate. However, TCC adsorption capacities were higher at lower NiFe_2_O_4_@C-900 dose which is described by Equation (2). Finally, the optimum solutions for adsorption of CFX were proposed as follows, concentration of 40.0 mg/L, adsorbent weight of 0.148 g/L, and pH 3.97. Meanwhile, the optimum solutions for adsorption of TCC were proposed as follows, concentration of 23.93 mg/L, adsorbent weight of 0.115 g/L, and pH 3.6. The desirability values were found at 0.885 and 1.0000 to obtain the highest capacities for CFX and TCC at 256.244, and 105.38 mg/g, respectively.

Model verification was used to repeat the proposed conditions, and attain the statistical errors of experimental capacity values. As expected, the verified errors were lower than 5.0%, suggesting the good compatibility of the quadratic models proposed in this study.

### 3.4. Recyclability Study

**Figure 13:**
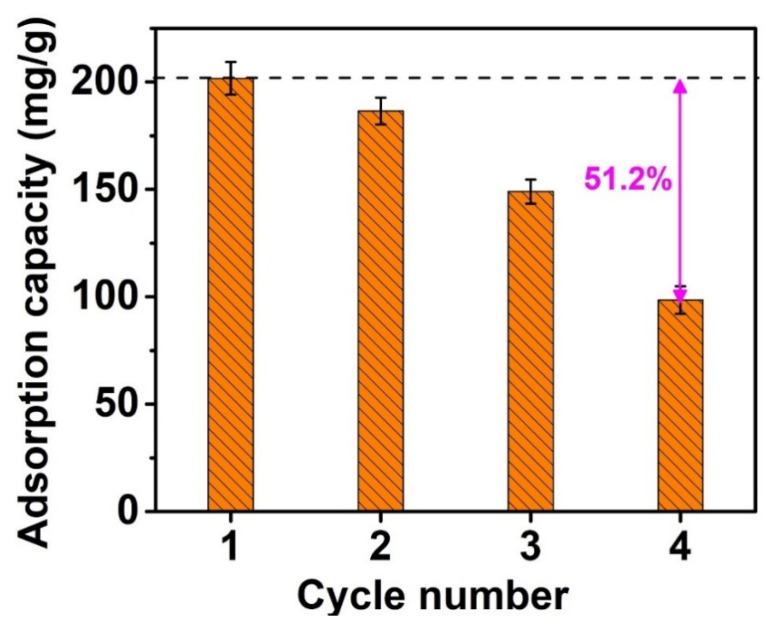
Reusability experiment for CFX adsorption on NiFe_2_O_4_@C-900. Experimental conditions included contact time: 180 min, dose: 0.1g/L, initial concentration (CFX and TCC): 20–30 mg/L, temperature: 30 °C, pH: 4 (for CFX) and 3 (for TCC), repeatability (*n* = 3) and error bars for all experiments.

The research on material recyclability are an important strategy to demonstrate the potential effect of sustainable adsorbents on environmental remediation. This experiment was carried out under the following conditions: NiFe_2_O_4_@C-900 material weight of 0.15 g/L, CFX antibiotic concentration of 40 mg/L, time of 180 min, and pH 4. HCl solvent (0.1 mol/L in ethanol) was used as an effective eluent by referring to the study reported by [57]. Indeed, HCl in aqueous solution disassociates a species of Cl^−^. As a result, it can complete the adsorption with CFX, enhancing the effectiveness of desorption of CFX from NiFe_2_O_4_@C-900 material. Accordingly, Figure 13 shows that NFO NiFe_2_O_4_@C-900 could be reused at least three times with a moderate change (~25%) in adsorption capacity, from 201.75 (1st run) to 149.0 mg/g (3rd run). However, there was a considerable decrease in adsorption (98.5 mg/g) for 4th run with 51.2%. Thereby, NiFe_2_O_4_@C-900 may be a potential material for antibiotic adsorption.

## 4. Conclusions

In this work, a series of NiFe_2_O_4_@C-x composites were successfully produced from a bimetallic-based metal-organic framework Ni-MIL-88B(Fe). Production temperatures (600–900 °C) had a strong effect on the structure and adsorption properties of CFX and TCC antibiotics. The characteristics indicated the existence of NiFe_2_O_4_ nanoparticles in the carbon matrix/layer. The NiFe_2_O_4_@C-x composites possessed a range of functional groups. For adsorption results, the composite produced at 900 °C obtained higher adsorption capacities for both CFX and TCC. The optimum pH investigations were achieved at pH 4 for CFX and pH 3 for TCC. The other optimum conditions of NiFe_2_O_4_@C-900 dose, contact time, and concentration were found, at 0.1 g/L, 180 min, and 20–30 mg/L, respectively. Response surface methodology was applied for quadratic models with very high coefficient of determination and adequate precision, suggesting high compatibility with experimental data. For CFX adsorption, the experimental conditions were identified at concentration of 40.0 mg/L, adsorbent weight of 0.148 g/L, and pH 3.97. Additionally, for TCC adsorption, the experimental conditions were identified at concentration of 23.93 mg/L, adsorbent weight of 0.115 g/L, and pH 3.6. The desirability values were found, at 0.885 and 1.0000, respectively. Under such proposed optimum conditions, the experimental adsorption capacities for CFX and TCC were found at 256.244 and 105.38 mg/g, respectively. Recyclability study indicated NiFe_2_O_4_@C-900 could be reused up to three cycles, offering the potential of this composite adsorbent for removal of emergent antibiotics.

## Figures and Tables

**Table 1:** Box–Behnken designs for main factors with value range.

No	Independent Factor	Unit	Code	Value Range					
				TCC			CFX		
				–1	0	+1	–1	0	+1
1	Antibiotic concentration	mg/L	x_1_	10	20	30	20	30	40
2	Adsorbent dosage	g/L	x_2_	0.05	0.1	0.15	0.05	0.1	0.15
3	pH	–	x_3_	2	3	4	3	4	5

**Table 2:** A summary of kinetic constants for adsorption of CFX and TCC on NiFe_2_O_4_@C-900.

Models	Parameter	Unit	TCC	CFX
Pseudo first–order	k_1_	min^–1^	0.2557	0.1555
	Q_1_	mg/g	149.47	151.88
	R^2^	–	0.9917	0.9785
Pseudo second–order	k_2_	g/(mg·min)	0.0057	0.00205
	Q_2_	mg/g	151.99	157.64
	R^2^	–	0.9951	0.9854
Elovich	α	mg/(g.min)	7.7 × 10^10^	1.54 × 10^5^
	β	g/mg	0.187	0.094
	R^2^	–	0.9975	0.9787
Bangham	k_B_	mL/(g/L)	126.36	108.12
	α_B_	–	0.0367	0.0719
	R^2^	–	0.9975	0.9773

**Table 3:** A summary of isotherm constants for adsorption of CFX, and TCC on NiFe_2_O_4_@C-900.

Models	Parameter	Unit	TCC	CFX
Langmuir	k_L_	L/mg	0.0281	0.0542
	Q_m_	mg/g	827.34	737.42
	R^2^	–	0.9218	0.9499
Freundlich	k_F_	(mg/g)/(mg/L)^1/n^	42.43	75.16
	1/n	–	0.623	0.506
	R^2^	–	0.9040	0.8711
Temkin	k_T_	L/mg	0.4035	0.4549
	B_T_	–	150.81	174.56
	R^2^	–	0.8542	0.9714

**Table 4:** Experimental and predicted adsorption capacity vales for the adsorption models of CFX and TCC on NiFe_2_O_4_@C-900.

No	CFX					TCC				
	Factors			Actual (mg/g)	Predicted (mg/g)	Factors			Actual (mg/g)	Predicted (mg/g)
	x_1_	x_2_	x_3_			x_1_	x_2_	x_3_		
1	20	0.05	3	35	33.21	10	0.05	2	49	47.49
2	40	0.05	3	115	124.09	30	0.05	2	83	89.67
3	20	0.15	3	98	94.53	10	0.15	2	53	57.35
4	40	0.15	3	195	207.05	30	0.15	2	74	77.02
5	20	0.05	5	92	95.65	10	0.05	4	55	53.16
6	40	0.05	5	169	188.17	30	0.05	4	90	86.83
7	20	0.15	5	79	85.61	10	0.15	4	86	80.51
8	40	0.15	5	182	199.63	30	0.15	4	89	91.69
9	13.2	0.1	4	81	85.68	3.2	0.1	3	29	32.24
10	46.8	0.1	4	285	258.11	36.8	0.1	3	82	77.10
11	30	0.016	4	150	139.74	20	0.016	3	86	86.48
12	30	0.184	4	213	201.06	20	0.184	3	101	102.71
13	30	0.1	2.3	35	33.07	20	0.1	1.3	75	68.12
14	30	0.1	5.7	100	79.59	20	0.1	4.7	80	85.21
15	30	0.1	4	196	205.80	20	0.1	3	102	102.71
16	30	0.1	4	221	205.80	20	0.1	3	105	102.71
17	30	0.1	4	210	205.80	20	0.1	3	103	102.71
18	30	0.1	4	188	205.80	20	0.1	3	102	102.71
19	30	0.1	4	218	205.80	20	0.1	3	101	102.71
20	30	0.1	4	198	205.80	20	0.1	3	103	102.71

**Table 5:** ANOVA data for the adsorption models of CFX and TCC on NiFe_2_O_4_@C-900.

Sources	Sum of Squares	Freedom Degree	Mean Square	F-Value	Prob. > F	Remarks
CFX model	87,334.80	9	9703.87	29.74	<0.0001	Significant
x_1_	35,888.21	1	35,888.21	109.98	<0.0001	–
x_2_	4538.20	1	4538.20	13.91	0.0039	–
x_3_	2596.73	1	2596.73	7.96	0.0181	–
x_1_ x_2_	231.13	1	231.13	0.7083	0.4197	–
x_1_ x_3_	1.13	1	1.13	0.0034	0.9543	–
x_2_ x_3_	2556.13	1	2556.13	7.83	0.0188	–
(x_1_)^2^	2070.67	1	2070.67	6.35	0.0304	–
(x_2_)^2^	2257.94	1	2257.94	6.92	0.0251	–
(x_3_)^2^	40,210.20	1	40,210.20	123.22	<0.0001	–
Residuals	3263.20	10	326.32	–	–	–
Lack of Fit	2394.37	5	478.87	2.76	0.1451	Not significant
Pure Error	868.83	5	173.77	–	–	–
Core Total	90,598.00	19	–	–	–	–
Fitness statistics: standard deviation (SD = 18.06), mean = 153.0, coefficient of variation (CV = 11.81%), coefficient of determination (R^2^ = 0.9640), adequate precision (AP = 17.6179).						
TCC model	8412.03	9	934.67	37.57	<0.0001	Significant
x_1_	2429.05	1	2429.05	97.64	<0.0001	
x_2_	184.72	1	184.72	7.43	0.0214	
x_3_	352.76	1	352.76	14.18	0.0037	
x_1_ x_2_	253.12	1	253.12	10.18	0.0097	
x_1_ x_3_	36.12	1	36.12	1.45	0.2559	
x_2_ x_3_	153.13	1	153.13	6.16	0.0325	
(x_1_)^2^	4158.80	1	4158.80	167.18	<0.0001	
(x_2_)^2^	181.88	1	181.88	7.31	0.0222	
(x_3_)^2^	1222.28	1	1222.28	49.13	<0.0001	
Residual	248.77	10	24.88			
Lack of fit	239.43	5	47.89	25.65	0.0014	Significant
Pure error	9.33	5	1.87			
Core total	8660.80	19				
Fitness statistics: standard deviation (SD = 4.99), mean = 82.40, coefficient of variation (CV = 6.05%), coefficient of determination (R^2^ = 0.9713), adequate precision (AP = 19.9834).						

## Data Availability

The data presented in this study are available on request from the corresponding author.
